# Plants obey (and disobey) the island rule

**DOI:** 10.1073/pnas.1907424116

**Published:** 2019-08-19

**Authors:** M. Biddick, A. Hendriks, K. C. Burns

**Affiliations:** ^a^School of Biological Sciences, Victoria University of Wellington, Wellington 6012, New Zealand

**Keywords:** functional trait, insular dwarfism, insular gigantism, size diversity, size evolution

## Abstract

The island rule predicts that small animals evolve to become larger on islands, while large animals evolve to become smaller. It has been studied for over half a century, and its validity is fiercely debated. Here, we provide a perspective on the debate by conducting a test of the island rule in plants. Results from an extensive dataset on islands in the southwest Pacific illustrate that plant stature and leaf area obey the island rule, but seed size does not. Our results indicate that the island rule may be more pervasive than previously thought and that support for its predictions varies among functional traits.

The island rule, a graded trend from gigantism in small species to dwarfism in large species on islands, is a controversial issue in biogeography (1, 2). While many studies have found support for its predictions (2–7), other studies have not (8–13), leading to widespread debate over its validity (14). Although studied for over 50 y, previous tests have been limited to animals, and predominantly to particular groups of vertebrates.

Mechanistically, several factors are thought to drive the convergence of body size on islands (3). For example, competition in species-rich mainland communities is thought to drive phenotypic divergence in order to promote coexistence (15). On islands, which tend to be more species-poor than mainlands, selection for phenotypic divergence is relaxed, leading to reduced size diversity. Given that these factors are not exclusive to animals, they might drive convergence in the size of other life groups.

Darwin (16) noted that many island trees are derived from continental herbs. He reasoned that herbaceous plants evolve woodiness on islands because of selection for increased stature, which improves their capacity to compete for light. Molecular tools have since demonstrated the convergent evolution of woodiness in the Canary (17, 18), Madeiran (19), and Hawaiian (20) floras. However, a unidirectional evolutionary pathway toward insular woodiness (and consequently increased stature) is not always observed (21, 22), and no previous study has tested for the island rule in plants.

We provide a test of the island rule in plants. We collected data on plant stature, leaf area, and seed size in 175 taxonomic pairings inhabiting 10 isolated archipelagos. Data were derived from field measurements, herbarium specimens, and flora descriptions from islands spanning 13 degrees of latitude of the southwest Pacific, to test whether small plants evolve to become larger on islands and large plants evolve to become smaller.

## Results

**Fig. 1.**
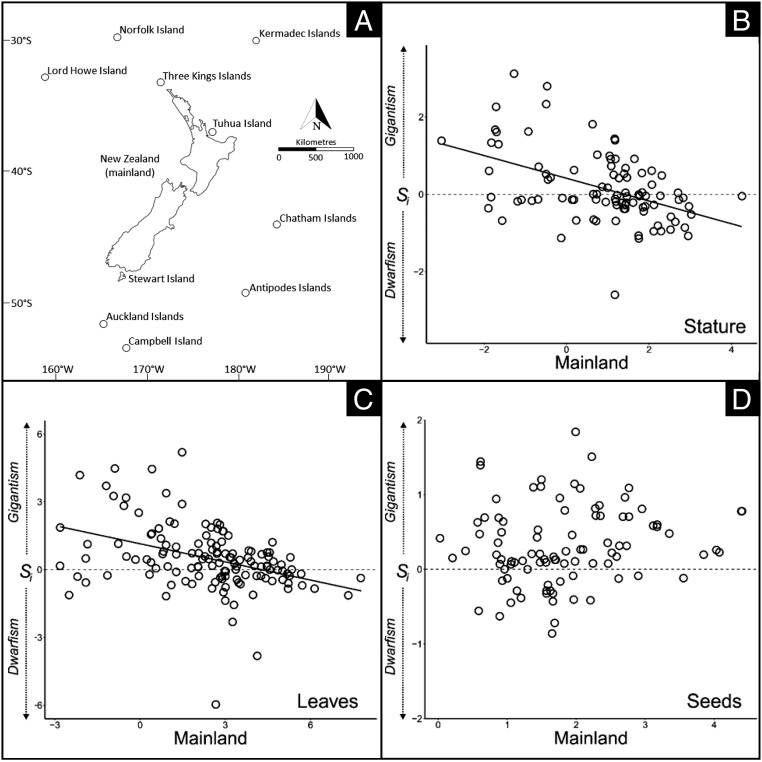
(*A*) The island rule was tested in plants inhabiting islands in the southwest Pacific, whose floras are primarily derived from the New Zealand “mainland.” (*B* and *C*) Insular size changes (*S*_*i*_, *y* axis) vary as a function of mainland values (*x* axis) in (*B*) stature (*n* = 96) and (*C*) leaves (*n* = 134). (*D*) Changes in seed size are unrelated to mainland values (*n* = 94). A dashed horizontal line intercepting the *y* axis at zero denotes morphological isometry. Both axes are logarithm-transformed. Open circles denote single island–mainland pairings.

We compiled 175 taxonomic pairings from 10 archipelagos surrounding the New Zealand “mainland” (Fig. 1*A* and Dataset S1). Linear regression revealed a graded trend from gigantism to dwarfism in both stature (Fig. 1*B*; *T* = −5.097, degree of freedom [*df*] = 93, *P* < 0.001) and leaf area (Fig. 1*C*; *T* = −4.910, *df* = 131, *P* < 0.001). Mixed effects models confirmed that these trends were robust after controlling for degree of taxonomic differentiation, growth form, collection method, and phylogenetic morphological conservatism (*T* = −6.131, *P* = 0.026; *T* = −4.044, *P* < 0.001, respectively). Paired *t* tests revealed that island values of stature and leaf area were not consistently larger or smaller than mainland values (*T* = 0.271, *df* = 95, *P* = 0.787; *T* = 0.226, *df* = 132, *P* = 0.821, respectively). Conversely, changes in seed size were ungraded (Fig. 1*D*; *T* = 0.994, *df* = 92, *P* = 0.333) even after controlling for potentially confounding factors (*T* = 0.778, *P* = 0.444). Island seed sizes were, instead, predominantly larger than mainland seed sizes (*T* = 4.051, *df* = 93, *P* < 0.001).

## Discussion

Our findings add to a growing body of evidence suggesting that plants, like animals, evolve in consistent ways on islands (23). Many previous studies on animals have documented support for the island rule (3–7, 24, 25), while others have failed to find support for its predictions (8–12). Here, we show that plants both obey and disobey the island rule, depending on the plant functional trait in question.

Plant stature and leaf area both obey the island rule. Therefore, they may have a single mechanistic explanation, if one trait covaries allometrically with the other. Previous work on animals has linked the island rule to a variety of factors, including insular changes in competitors, predators, or environmental conditions (3, 4). The same ecological mechanisms could drive the evolutionary convergence of size in island plants. On the other hand, given the physiological differences between animals and plants, other processes might be at work.

This would not appear to be the case with seed size, as it disobeys the island rule. Instead, it exhibits a consistent tendency toward gigantism, a phenomenon that has been documented elsewhere and is thought to arise for reasons related to dispersal ability (i.e., reduced mortality at sea, ref. 16, but see ref. 26). Alternatively, islands house fewer species at greater densities than mainlands (27). Therefore, a selection for larger (and consequently more competitive) seeds could arise from greater levels of competition among conspecifics.

Future work on island plants may provide a unique window into the processes responsible for the island rule. Plants can be collected, transported, and manipulated more easily than animals. They can be grown under different environmental conditions, subjected to different herbivores in cafeteria-style experiments, and planted in competitive arrays. Therefore, future tests of the island rule in plants may help inform the debate over whether animals obey (or disobey) the island rule.

## Methods and Materials

### Data Collection.

We integrated data from published literature, flora descriptions, herbarium specimens, and field measurements (Dataset S1). We extracted data from 4 studies that share similar methodologies and were carried out by the same working group (28–31). These studies predominantly investigated size changes in taxonomically undifferentiated and partially differentiated island–mainland pairings. To include more taxonomically differentiated taxa, we used published molecular phylogenies to identify island endemics in the southwest Pacific that result from anagenesis following a single colonization event. When no phylogeny was available, geographic proximity was used as a surrogate for genetic relatedness. Stature, leaf area, and seed size values were then extracted from flora descriptions. We systematically extracted the greatest value for stature and the mean value for leaves and seeds. When only a single metric of size was available (e.g., length without width), the same metric was extracted for the respective comparison, such that trait metrics were always kept consistent within pairings. When trait values were unavailable, images of specimens were sourced from online herbaria and measured in ImageJ (32). Field measurements of a further 13 pairings from Tuhua Island were collected following the methodology of Biddick et al. (28). Mainland measurements were taken from the Kaimai-Mamaku Forest Reserve, which occupies the same ecological district and latitudinal band as Tuhua Island.

### Data Analysis.

Following Lomolino et al. (4), we first performed linear regressions of log(*S*_*i*_ [island value divided by mainland value]) against log(*M* [mainland value]). Paired *t* tests were then used to test whether island values were consistently larger or smaller than mainland values. We utilized linear mixed effects models to control for factors that might obscure island rule trends. Because *S*_*i*_ should vary with degree of taxonomic differentiation, we included taxonomic differentiation as a fixed effect with 3 levels (fully differentiated, partially differentiated, and undifferentiated). The partially differentiated level included both subspecies and varieties. Because *M* values should differ between woody and herbaceous plants, we included growth form as a fixed factor with 2 levels (woody and nonwoody). Species occur multiple times in the dataset; therefore, species identity was included as a random effect. To control for phylogenetic morphological conservatism, taxonomic family was included as a random effect. To control for variation related to collection method, collection method was included as a random effect.

## Supplementary Material

Supplementary File
